# Supplementary material for: Three-Dimensional-Bioprinted Embedded-Based Cerebral Organoids: An Alternative Approach for Mini-Brain In Vitro Modeling Beyond Conventional Generation Methods
Source: Gels. 2025 Apr 11;11(4):284. doi: 10.3390/gels11040284 (PMC12027382; doi:10.3390/gels11040284)
Supplement: Supplementary file 1 [file gels-11-00284-s001.zip › gels-3540625-supplementary.pdf]

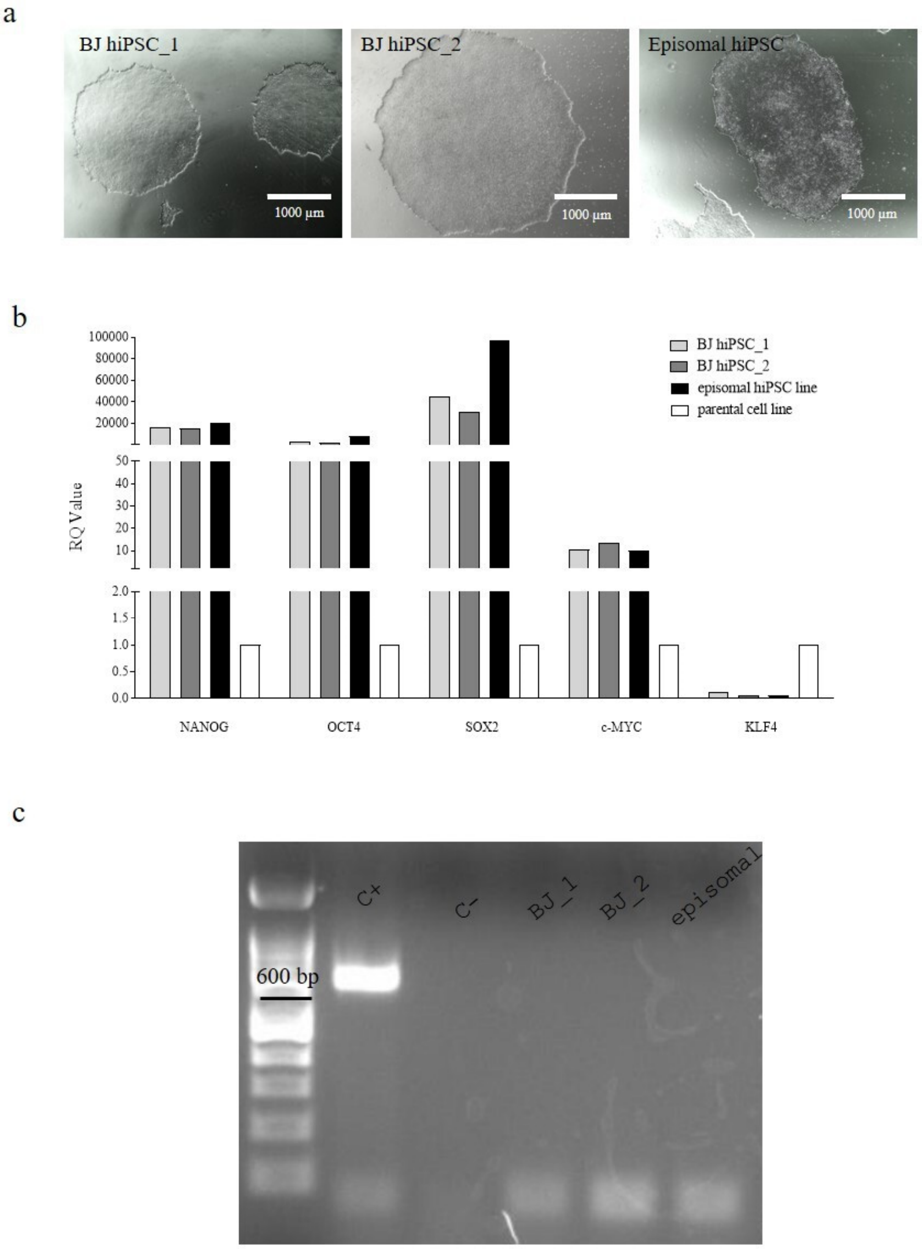

Figure S1. (**a**) iPSC clones morphology, (**b**) Gene expression analysis of pluripotency markers, (**c**) Mycoplasma detection.
